# Key Considerations for Studying the Effects of High-Fat Diet on the Nulligravid Mouse Endometrium

**DOI:** 10.1210/jendso/bvae104

**Published:** 2024-05-25

**Authors:** Hilary J Skalski, Amelia R Arendt, Shannon K Harkins, Madison MacLachlan, Cody J M Corbett, Robinson W Goy, Amita Kapoor, Galen Hostetter, Ronald L Chandler

**Affiliations:** Department of Obstetrics, Gynecology and Reproductive Biology, College of Human Medicine, Michigan State University, Grand Rapids, MI 49503, USA; Department of Obstetrics, Gynecology and Reproductive Biology, College of Human Medicine, Michigan State University, Grand Rapids, MI 49503, USA; Department of Obstetrics, Gynecology and Reproductive Biology, College of Human Medicine, Michigan State University, Grand Rapids, MI 49503, USA; Department of Obstetrics, Gynecology and Reproductive Biology, College of Human Medicine, Michigan State University, Grand Rapids, MI 49503, USA; Wisconsin National Primate Research Center, Assay Services, University of Wisconsin-Madison, Madison, WI 53715, USA; Wisconsin National Primate Research Center, Assay Services, University of Wisconsin-Madison, Madison, WI 53715, USA; Wisconsin National Primate Research Center, Assay Services, University of Wisconsin-Madison, Madison, WI 53715, USA; Pathology and Biorepository Core, Van Andel Research Institute, Grand Rapids, MI 49503, USA; Department of Obstetrics, Gynecology and Reproductive Biology, College of Human Medicine, Michigan State University, Grand Rapids, MI 49503, USA; Department for Epigenetics, Van Andel Research Institute, Grand Rapids, MI 49503, USA

**Keywords:** uterus, endometrium, obesity, mouse model, estrous cycle, ovary

## Abstract

The obesity epidemic continues to increase, with half of US women predicted to be obese by 2030. Women with obesity are at increased risk for not only cardiovascular and liver disease, but also reproductive disorders. Although mouse models are useful in studying the effects of obesity, there is inconsistency in obesity-induction methods, diet composition, and mouse strains, and studies using female mice are limited. In this study, we sought to compare the effects of a 45% high-fat diet (HFD) versus a 60% HFD on the uterine estrous cycle of nulligravid C57BL/6J mice. For 22 weeks, we placed a total of 20 mice on either a 60% HFD, 45% HFD, or each HFD-matched control diet (CD). Both HFDs produced significant weight gain, with 60% HFD and 45% HFD gaining significant weight after 2 weeks and 15 weeks, respectively. Additionally, both HFDs led to glucose intolerance, fatty liver, and adipocyte hypertrophy. Mice fed 60% HFD displayed hyperphagia in the first 12 weeks of HFD treatment. Moreover, 60% HFD-treated mice had a longer estrous cycle length and an increased percentage of estrus stage samplings compared to CD-treated mice. Estrous cycle stage-controlled 60% HFD-treated mice displayed an increased estrogen-to-progesterone ratio and decreased ovarian corpora lutea compared to CD-treated mice, which may underlie the observed estrous cycle differences. There was no significant difference between diets regarding endometrial morphology or the percent of endometrial CD45+ immune cells. Our results indicate that consideration is needed when selecting a HFD-induced obesity mouse model for research involving female reproductive health.

Obesity, defined as a body mass index (BMI) of 30 or greater, is an increasing epidemic within the United States. It is predicted that roughly 50% of adult women in the United States will be obese by the year 2030 [1]. Obesity in women is associated with increased estrogen unopposed by progesterone. This rise in estrogen is, in part, due to increased extragonadal aromatization of androgens into estrogens in women with obesity [2, 3]. This hormonal imbalance can lead to menstrual irregularities, a 2.7-fold decrease in fertility, as well as reproductive pathologies, including endometrial hyperplasia and its malignant counterpart, endometrial cancer [4-6].

Mouse models of obesity are either genetically-induced obese models, like *ob/ob* or *db/db*, or diet-induced obese (DIO) models. While *ob/ob* and *db/db* mice are effective in producing obese mice, the genetic mutations driving this phenotype are not translationally relevant to human obesity, as few humans have these genetic mutations [7-9]. Alternatively, DIO mouse models can be complicated as there is not consensus on the ideal macro- and micronutrient concentrations to stimulate murine obesity. Most commonly, fat percentages are altered in these studies, with high-fat diets (HFD) typically ranging from 45% to 60% fat, while control diets (CDs) contain ∼10% fat [10, 11]. It is essential to control for additional variables when selecting a matching CD. Some studies use grain-based control diets, which fail to limit excess phytoestrogens and can cause variability between diet batches [12-16]. Additionally, CDs do not always contain sucrose- and micronutrient-matched profiles with their chosen HFD [10]. Which mouse strain is utilized for an obesity study is also important, as some mouse models are more resistant to obesity. For example, the BALB/c mouse is much more resistant to obesity when compared to the C57BL/6J mouse [17].

The majority of recent studies investigating the effects of diet-induced obesity do so only on male mice [11]. In contrast to C57BL/6J male mice, female C57BL/6J mice have slower weight gain on a 60% HFD and have an increased glucose tolerance compared to their male counterparts, especially when the diet was initiated just after sexual maturation at 6 weeks old [17-19]. Therefore, due to the relative scarcity of research in obese female mice, lack of consensus in fat concentrations, and the limited use of nutrient-matching and phytoestrogen-limiting control diets, it is vital to investigate the impact of 45% and 60% HFDs in relation to their respective purified and micronutrient-matched CDs in female C57BL/6J mice. In this study, we utilized Research Diets, Inc. in-stock DIO series of HFDs due to its prevalence in other studies, purified vs grain-based ingredients, micronutrient (sucrose, vitamin, and mineral)-matched CDs, and formulations which minimize phytoestrogens. The aim of this study is to investigate the impact of 45% and 60% HFDs on obesity-associated characteristics and, more specifically, uterine histology and cyclicity in female C57BL/6J mice, with the intention of future studies focusing on the effects of obesity on the endometrium. These results will provide guidance to researchers performing a HFD-induced obesity experiment in female mice.

## Methods

### Mice

Four-week-old female C57BL/6J wild-type (WT) mice were purchased from The Jackson Laboratory (Strain #000664, Bar Harbor, ME, USA) and given 3 weeks to acclimate before initiating diets. In the first cohort of mice, 7-week-old mice were put on either a 60% HFD (Research Diets, D12492) or its respective 10% low-fat (7% sucrose-matched) CD (Research Diets, D12450J) or a 45% HFD (Research Diets, D12451) or its respective 10% low-fat (17% sucrose-matched) CD (Research Diets, D12450H). The second cohort of mice started diet treatments at 5 to 12 weeks of age and were only given either the 60% HFD or the 60% CD. Mice were fed *ad libitum* for 22 weeks in the first cohort and 30 to 37 weeks in the second cohort. Mice were housed on a 24-hour light cycle, with light-phase from 7 Am (Zeitgeber time; ZT0) to 7 Pm (ZT12) and dark-phase from 7 Pm (ZT12) to 7 Am (ZT0). Cage changes were performed weekly. Mouse body weight and amount of food consumed were measured weekly. To determine the estimated average amount of food eaten per mouse each week, the amount of food eaten per cage each week was divided by the number of mice in the cage. For the first cohort of mice, starting at 19 weeks on diet, estrous cycle length was assessed via daily vaginal lavage over a 2-week period. At 19 to 22 weeks on the diet, a glucose tolerance test was performed. On the day of tissue harvest, estrous cycle stage was obtained. Blood was collected from the superficial temporal vein via check bleed for serum hormone analysis. Mice were sacrificed via CO_2_ inhalation after 22 weeks on the diet (30 weeks of age) in the first cohort and specifically during the estrus stage of the estrous cycle after 30 to 37 weeks on the diet (35-43 weeks of age) for the second cohort. Uterus, ovaries, adipose tissue, liver, and kidney sections were placed in either 4% paraformaldehyde (EMS, Ref#15710) or 10% neutral-buffered formalin (VWR, Ref#16004-115) at the time of tissue harvest. Mice were housed at Michigan State University's (MSU) Grand Rapids Research Center in accordance with protocols approved by MSU's Institutional Animal Care and Use Committee (IACUC). MSU is registered with the US Department of Agriculture (USDA) and has an approved Animal Welfare Assurance from the National Institutes of Health Office of Laboratory Animal Welfare (OLAW). MSU is accredited by the Association for Assessment and Accreditation of Laboratory Animal Care (AAALAC).

### Estrous Cycle Evaluation

To determine the estrous cycle stage, after 19 weeks on diet, vaginal lavage was performed on the mice at the same time daily for 2 weeks. Vaginal lavage and cytology were additionally performed on the morning of tissue harvest. Cells were collected vaginally by pipetting sterile deionized water in and out of the vaginal canal 10 times before placing the fluid collection onto a slide to dry. Once the sample dried, the slide was fixed with methanol, then stained with concentrated Wright-Giemsa stain (Azer Scientific, Ref#ES921), followed by Wright-Giemsa stain diluted in 1:5 phosphate buffered saline (PBS) (pH 6.8), and then rinsed with sterile deionized water. Slides were then dried and subsequently dehydrated in 95% ethanol, 100% ethanol, and CitriSolv® (Decon Labs, Ref#1601) before being cover slipped and cells viewed under a light microscope for staging. Daily estrous cycle stage and final estrous cycle stage (at tissue harvest) were determined by the relative concentrations of nucleated epithelial cells, cornified epithelial cells, and leukocytes [20-22]. Average cycle length for each mouse was computed and then used for the final graph. A state of intermittent estrous cycling was defined by a mouse having the same estrous cycle stage on daily vaginal lavage for a minimum of 6 days consecutively. Additionally, the number of samplings per cycle stage was summed for each diet to determine the percent of samplings in each estrous cycle stage by diet. This percentage was then used to investigate if the diet was correlated with the occurrence percentage of each estrous cycle stage.

### Glucose Tolerance Test

At 20 weeks on diet (27 weeks old), a glucose tolerance test was performed with fasting initiated at 7 Am (ZT0) and mouse weight recorded to calculate glucose injections. The glucose concentration time course began at 1 Pm (ZT6) after 6 hours of fasting. At this time (timepoint 0, ZT6), the tip of the tail was cut and massaged until a drop of blood was obtained for the fasting glucose level. Glucose levels were measured with a glucometer (One Touch Ultra 2 Blood Glucose Monitoring System) and test strips (True Point Generic Test Strips, Ref#64230). Mice were then immediately intraperitoneally injected with 2 g/kg of 0.2 g/mL autoclaved USP D(+)-Glucose (Millipore, Ref#1.37048) dissolved in 0.9% saline. Blood glucose levels were then additionally measured 30, 60, 90, 120, 150, 180, 210, and 240 minutes post-dextrose injection by massaging the tail to collect a drop of blood from the previous collection area.

### Serum Hormone Levels

Blood was collected through a cheek bleed on the day of tissue harvest. Blood was immediately mixed with dipeptidyl peptidase IV inhibitor (Millipore, Ref#DPP4) and a protease inhibitor cocktail (Sigma, Ref#P2714). Following collection, blood was left at room temperature for 30 minutes, then centrifuged at 4 °C and serum supernatant collected and stored at −80 °C until further use. Serum was later shipped to the Wisconsin National Primate Research Center's Assay Services Unit at the University of Wisconsin-Madison where liquid chromatography with tandem mass spectrometry was performed on the samples to obtain circulating estrone, 17β-estradiol, and progesterone levels. The method was adapted from that previously developed in the lab, but was conducted with a Sciex 6500 + triple quadrupole mass spectrometer [23].

### Histology and Immunohistochemistry

Ten percent neutral-buffered formalin-fixed tissues were paraffin embedded, sectioned, and hematoxylin and eosin (H&E) stained at the Pathology and Biorepository Core at Van Andel Research Institute (Grand Rapids, MI, USA). Adipocyte size was measured from a representative 10 × objective view of white adipose tissue H&E slides. Each adipocyte's size in the 10 × view was measured in pixels using the Adiposoft plug-in for ImageJ [24]. Average adipocyte size for each mouse was then calculated and relative fold change of adipocyte size was compared between the HFD and CD. H&E slides of the estrus-staged ovary were used to calculate the average largest ovarian area, as well as average ovarian follicle counts per histological section by diet type. Multiple serial sections, at a minimum of 20 µm apart, were measured per ovary to obtain the average count for each ovarian follicle type. The average number of primordial, primary, secondary, and antral follicles, corpora lutea (CL), and total number of follicles per section were measured [25, 26]. Additional sectioned slides of the uterus were then stained for various markers using indirect immunohistochemistry (IHC). Slides were deparaffinized with CitriSolv® (Decon Labs, Ref#1601), processed for heat-based antigen unmasking in 10 mM sodium citrate (pH 6.0), and incubated in 3% hydrogen peroxide. Blocking was performed with 5% Normal Donkey Serum (Jackson Immuno-Research Lab, Ref#017-000-121) in Tris-buffered saline with 0.1% Tween-20. Slides were incubated with primary antibodies diluted in SignalStain® Ab Diluent (Cell Signaling, Ref#8112) at the following dilutions: KRT8 1:100 (Developmental Sciences Hybridoma Bank, Ref#TROMA-I, RRID:AB_531826) and CD45 1:200 (Cell Signaling, Ref#70257, RRID:AB_2799780). Biotin-conjugated secondary antibodies were diluted with Animal-Free Blocking Solution (Cell Signaling, Ref#15019) at the following dilutions: 1:500 donkey anti-rat IgG (Jackson Immuno-Research Lab, Ref#712-065-153, RRID:AB_2315779) and 1:250 donkey anti-rabbit IgG (Jackson Immuno-Research Lab, Ref#711-065-152, RRID:AB_2340593). Slides were then incubated with VECTASTAIN® Elite® ABC Peroxidase Kit (Vector Laboratories, #RefPK-6100), developed with the ImmPACT® DAB Substrate Peroxidase Kit (Vector Laboratories, #RefSK-4105), post-fixed with 4% paraformaldehyde in PBS and counterstained with Hematoxylin QS (Vector Laboratories, Ref#H-3404). Lastly, slides were dehydrated and mounted with CitraMount™ Medium (Polysciences, Ref#24214-100). Every mouse was assayed for each marker. Images were scanned by the Van Andel Research Institute Pathology and Biorepository Core and viewed with Aperio ImageScope software. The percentage of CD45+ endometrial cells per diet was calculated from a representative gridded 20 × field of view from each animal. The average of the squares was taken to determine the average percentage of CD45+ cells per mouse. Final estrous cycle stage at tissue harvest was used to determine groupings for percent of CD45+ cells during each stage of the estrous cycle.

### Statistical Analysis

All statistical analyses were performed using GraphPad Prism (Version 10). Average weekly food consumed per mouse, final body weight, total weight gain, percent body weight gain, relative adipocyte area, uterus weight, uterus-to-body weight ratio, circulating hormone concentrations, ovarian area, follicle counts, estrous cycle length, and percent CD45+ endometrial cells were analyzed with a two-tailed unpaired *t*-test statistic or Welch's *t*-test statistic when there were unequal variances. Amount of food eaten each week between diets, weight gain each week between diets, glucose levels at each timepoint between diets, and percent of occurrences in each cycle stage between diets were analyzed with a two-tailed multiple unpaired Welch's *t*-test statistic. Percent of CD45+ cells during each stage of the estrous cycle was analyzed with Brown–Forsythe and Welch’s one-way ANOVA with Dunnett's T3 multiple comparisons test. The estimated average amount of food eaten each week per mouse was calculated by the total amount of food eaten per cage divided by the number of animals in the cage. All other data is represented as mean ± standard deviation (SD). The following refers to *P*-values denoted in figures: *n.s.*  *>* 0.05; *P < .05; **P  *<* .01; ***P  *<* .001; ****P  *<* .0001.

## Results

### Both 45% and 60% HFD Result in Increased Body Weight While Only 60% HFD Induces Hyperphagia

**Figure 1.**
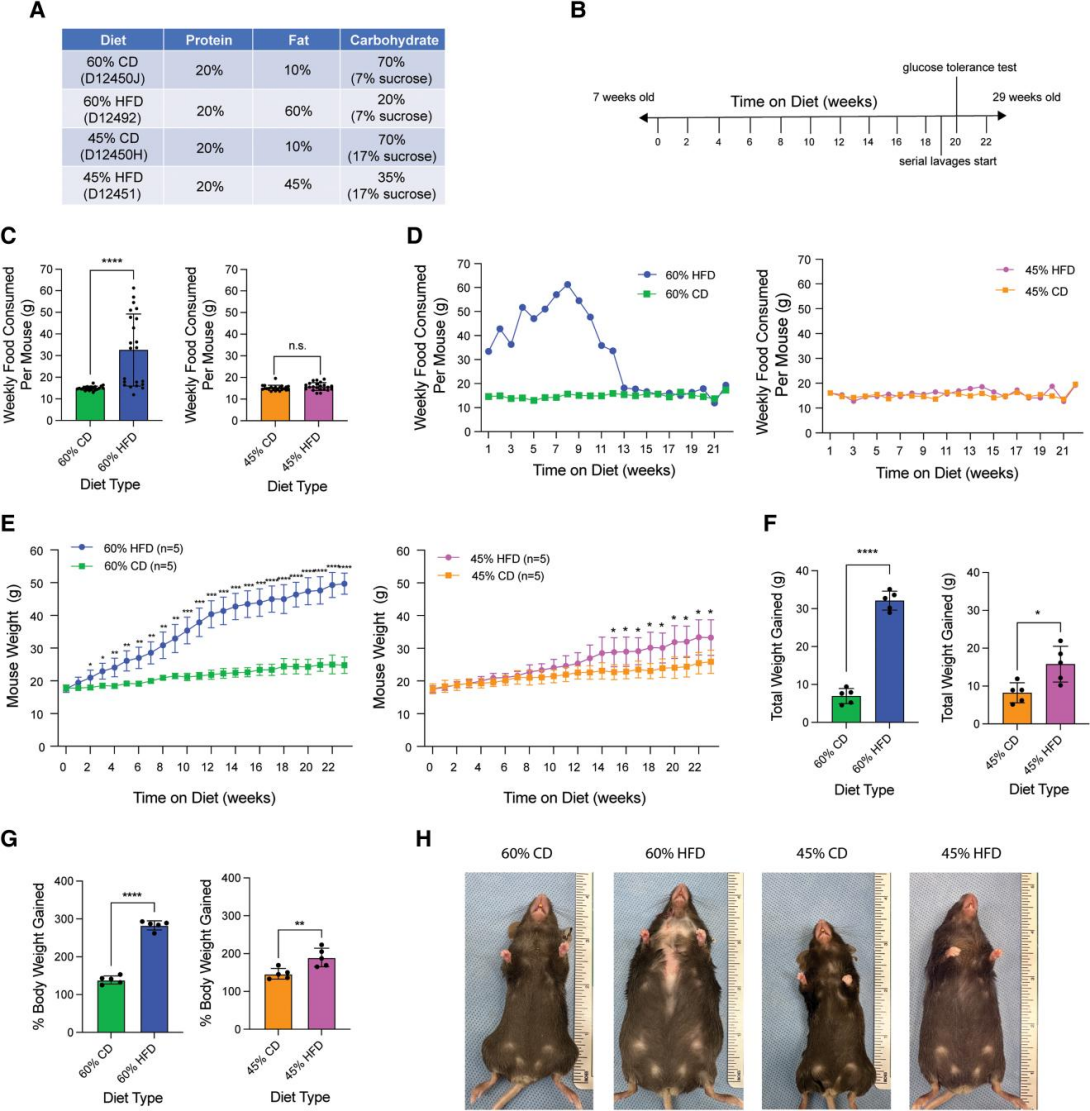
Both 45% and 60% HFD increase body mass compared to CD, but only 60% HFD induces hyperphagia. (A) Nutrient compositions of the 60% CD, 60% HFD, 45% CD, and 45% HFD from Research Diets, Inc. CDs are sucrose matched to their respective HFD, meaning if matched CD and HFD mice eat the same amount of calories, then they will ingest the same amount of sucrose. Macronutrient percentages are given in kcal%. (B) Experimental timeline. (C) Estimated average amount of food consumed per mouse per week (grams) for 60% HFD compared to 60% CD and 45% HFD compared to 45% CD. Measurements represent weekly (datapoint; n = 22) diet's mean ± SD. Two-tailed unpaired *t*-test statistic with Welch's correction. (D) Estimated average amount of food consumed (grams) per mouse separated per week over the course of the study. Weekly measurements (datapoint) represent the average amount of food consumed per mouse per diet for that week. Each treatment group filled only one cage, so average amount of food eaten per cage per week was divided by 5 for estimated average eaten per mouse per week, leaving all 5 mice in the condition with the same amount of food eaten. No statistical analysis was performed. (E) Average mouse body weight (grams) per diet over the course of the experiment. Measurements represent weekly (datapoint; n = 5 per condition) diet mean ± SD. Multiple unpaired *t*-tests statistic with Welch's correction. (F) Total amount of weight gained (grams) per mouse after 22 weeks on diet. Measurement (datapoint; n = 5 per condition) represents each mouse (bar = mean ± SD). Two-tailed unpaired *t*-test statistic. (G) Percent body weight gained by the end of the experiment (22 weeks on diet). Measurement (datapoint; n = 5 per condition) represents each mouse (bar = mean ± SD). Two-tailed unpaired *t*-test statistic. (H) Images of mouse body size after 22 weeks on diet. *P < .05; **P < .01; ***P < .001; ****P < .0001.

Abbreviations: CD, control diet; HFD, high-fat diet.

Both a 45% and 60% HFD were utilized on female C57BL/6J mice. There were two different CDs used in this study: one for controlling for the 60% HFD (which contained 7% sucrose in a total of 70% carbohydrates) and another for controlling for the 45% HFD (which contained 17% sucrose in a total of 70% carbohydrates). Therefore, if each HFD-treated mouse consumes the same number of calories as a respective CD-treated mouse, then they will ingest the same amount of sucrose. Percent protein remained consistent across diets, while carbohydrates were adjusted according to the change in fat. Macronutrient percentages are measured in kcal% (Fig. 1A). Mice were started on diet at 7 weeks of age and continued for 22 weeks, until 29 weeks of age (Fig. 1B). The 60% HFD mice exhibited hyperphagia, consuming significantly more food each week compared to the CD mice, while 45% HFD mice consumed amounts similar to their respective CD (Fig. 1C). When examining the amount of food ingested each week over the course of the study, the increased food consumption only occurred in the first half of the experiment (Fig. 1D). Baseline mouse body weight was 17.83 g ± 0.9524 g for 60% CD vs 17.57 g ± 1.099 g for 60% HFD (*P* = .699931) and 17.64 g ± 1.496 g for 45% CD vs 17.5 g ± 1.020 g for 45% HFD (*P* = .867578). Both 60% and 45% HFD mice gained significantly more weight compared to their CDs at 32.12 g ± 2.510 g (60% HFD) vs 6.95 g ± 2.003 g (60% CD) and 15.79 g ± 4.741 g (45% HFD) vs 8.22 g ± 2.648 g (45% CD). The two HFDs differed in the amount of time needed to achieve significant differences in body weight, with significant weight gain occurring after 2 weeks on the 60% HFD and after 15 weeks on the 45% HFD. The average final body weights (after 22 weeks on diet) were 24.78 g ± 2.487 g for 60% CD, 49.69 g ± 3.239 g for 60% HFD, 25.86 g ± 3.547 g for 45% CD, and 33.29 g ± 5.443 g for 45% HFD (Fig. 1E-1H).

### HFD Mice Display Obesity-Associated Comorbidities

**Figure 2.**
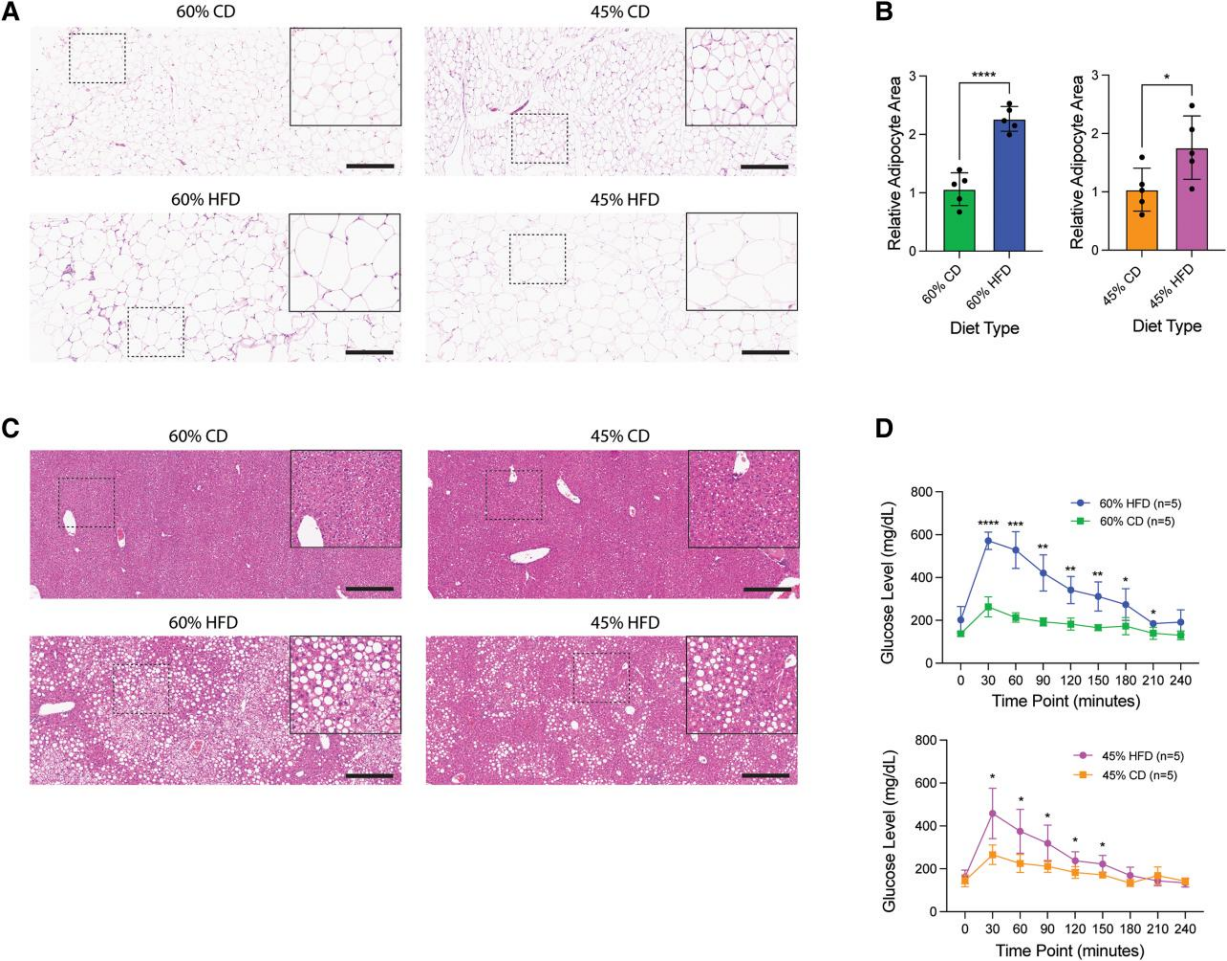
Both 45% and 60% HFD show adipocyte hypertrophy, fatty liver infiltration, and glucose intolerance. (A) Representative H&E stain of mouse white adipose tissue for each diet type. Main image = 10 × magnification; inset = 20 × magnification; scale bar = 300 µm. (B) Quantified mean relative adipocyte area between diets (HFD normalized to CD) using the Adiposoft plug-in for ImageJ from 10 × H&E stain of white adipose tissue. Measurements (datapoint; n = 5 per condition) are each mouse's mean adipocyte area. Bar represents diet's mean ± SD. Two-tailed unpaired *t*-test statistic. (C) Representative H&E stain of mouse liver for each diet type. Main image = 10 × magnification; inset = 20 × magnification; scale bar = 300 µm. (D) Blood glucose measurements (mg/dL) at 30-minute intervals after a 2 g/kg glucose injection of mice at 20 weeks on diet (n = 5 per condition). Measurements represent diet's mean ± SD. Multiple unpaired *t*-tests statistic with Welch's correction. *P < .05; **P < .01; ***P < .001; ****P < .0001.

Abbreviations: CD, control diet; H&E, hematoxylin and eosin; HFD, high-fat diet.

Obesity is commonly associated with adipocyte hypertrophy, fatty liver infiltration, and glucose intolerance [27-30]. Following quantification of adipocyte surface area from adipose tissue H&E staining, we observed adipocyte hypertrophy in both 60% and 45% HFD compared to their respective CDs (Fig. 2A, B). Additionally, H&E staining of the liver displayed increased ectopic lipid accumulation, suggesting fatty liver infiltration in both HFDs (Fig. 2C). Next, we performed glucose tolerance tests by challenging 6-hour fasted CD and HFD mice with glucose, then measuring serum glucose levels over time. After 20 weeks on diet (27 weeks of age), both 45% and 60% HFD presented with glucose intolerance, though fasting glucose levels were normal (Fig. 2D).

### Similar Histology and Immune Cell Composition in the Endometrium Among Diets

**Figure 3.**
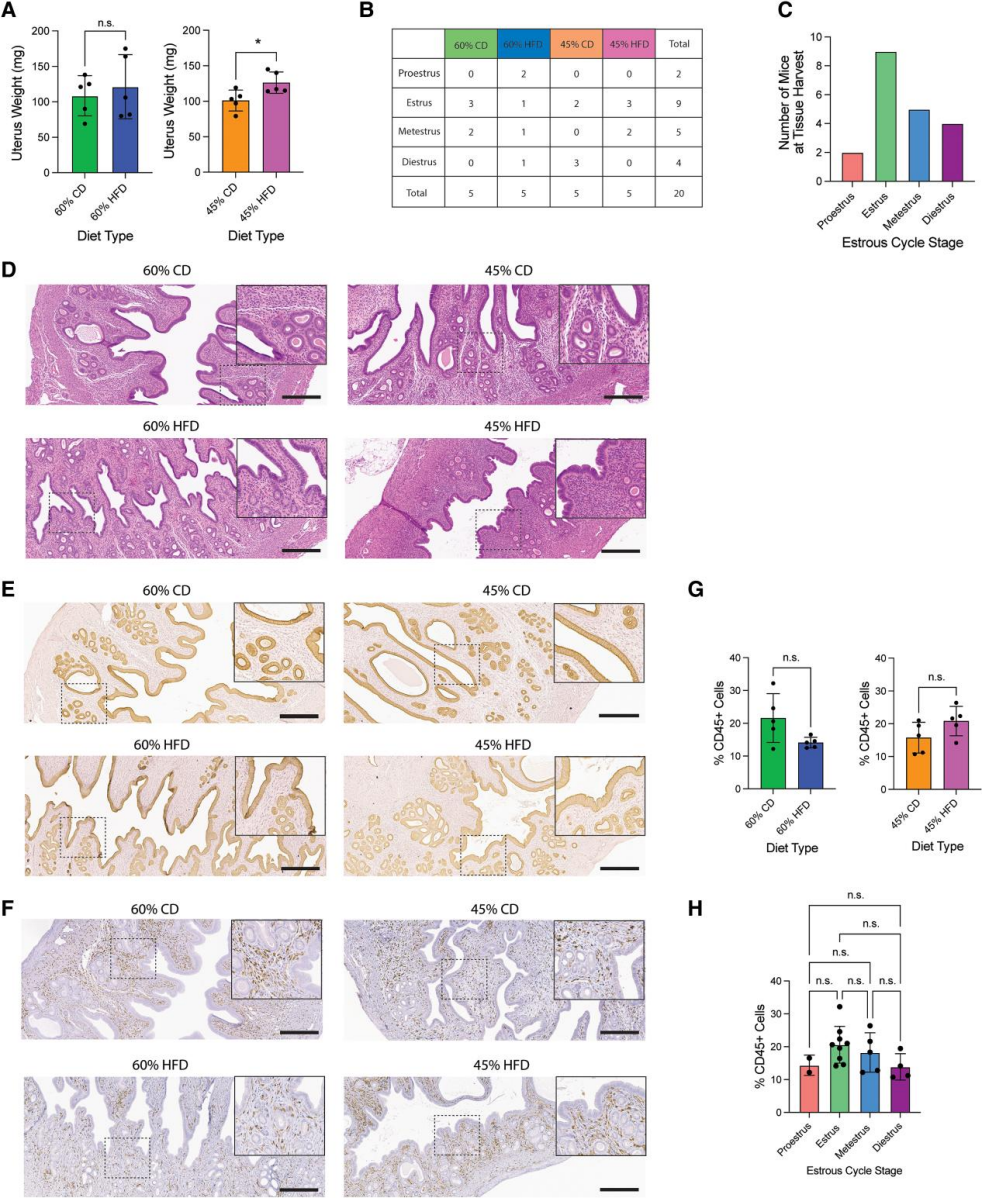
Normal uterine histology and comparable immune cell profiles across the diets. (A) Semi-dry uterine weight (mg) at tissue harvest after 22 weeks on diet. Measurement (datapoint; n = 5 per condition) represents each mouse (bar = mean ± SD). Two-tailed unpaired *t*-test statistic. (B) Table summarizing estrous cycle stage at tissue harvest by diet (n = 5 per condition). (C) Graph of mouse final estrous cycle stage at tissue harvest by diet. (D) Representative H&E stain of mouse endometrial tissue for each diet type during estrus. Main image = 10 × magnification; inset = 20 × magnification; scale bar = 300 µm. (E) Representative KRT8 (epithelial marker) IHC stain of mouse endometrial luminal and glandular epithelium for each diet type during estrus. Main image = 10 × magnification; inset = 20 × magnification; scale bar = 300 µm. (F) Representative CD45 (pan-immune cell marker) IHC stain of mouse endometrial tissue for each diet type during estrus. Main image = 10 × magnification; inset = 20 × magnification; scale bar = 300 µm. (G) Comparison of average percentage of CD45+ immune cells in a representative 20 × field of view of the endometrium between diets. Measurements (datapoint; n = 5 per condition) are each mouse's mean. Bar represents diet's mean ± SD. Two-tailed unpaired *t*-test statistic with Welch's correction. (H) Comparison of average percentage of CD45+ immune cells in a representative 20 × field of view of the endometrium comparing final estrous cycle stages. Measurements (datapoint) are each mouse's mean. Bar represents diet's mean ± SD. Brown–Forsythe and Welch’s one-way ANOVA and Dunnett's T3 multiple comparisons test statistics. *P < .05; **P < .01; ***P < .001; ****P < .0001.

Abbreviations: H&E, hematoxylin and eosin; IHC, immunohistochemistry.

After examining mice for common obesity-associated comorbidities, we shifted our focus to the female reproductive tract. First, we wanted to investigate the effect of HFD on the endometrium by histological examination. No gross pathological changes were apparent upon uterine dissection (not pictured). Surprisingly, semi-dry uterus weights of the 45% HFD uteri weighed significantly more than the 45% CD, though weights did not vary between the 60% HFD and its CD (Fig. 3A). This difference in uterine weight could be due to uteri not being estrous stage-matched, as uterine thickness varies throughout the estrous cycle [31]. Final estrous cycle stage was recorded for all mice on the day of tissue harvest (Fig. 3B-C). H&E uterine stains displayed morphological changes between endometrium of different estrous cycle stages, but no apparent endometrial histological differences between diets were observed (Fig. 3D). In addition to the H&E sections, we used IHC to stain for KRT8 (an epithelial marker) and CD45 (an immune cell marker). IHC staining for KRT8 showed relatively normal endometrial simple columnar luminal and glandular epithelia (Fig. 3E). The majority of CD45+ immune cells resided in the stroma of the endometrium, as compared to the luminal and glandular epithelium. There was no difference between diets or estrous cycle stage regarding the percent of CD45+ immune cells present in the endometrium (Fig. 3F-3H).

### 60% HFD Increases Estrous Cycle Length and Frequency of the Estrus Stage

**Figure 4.**
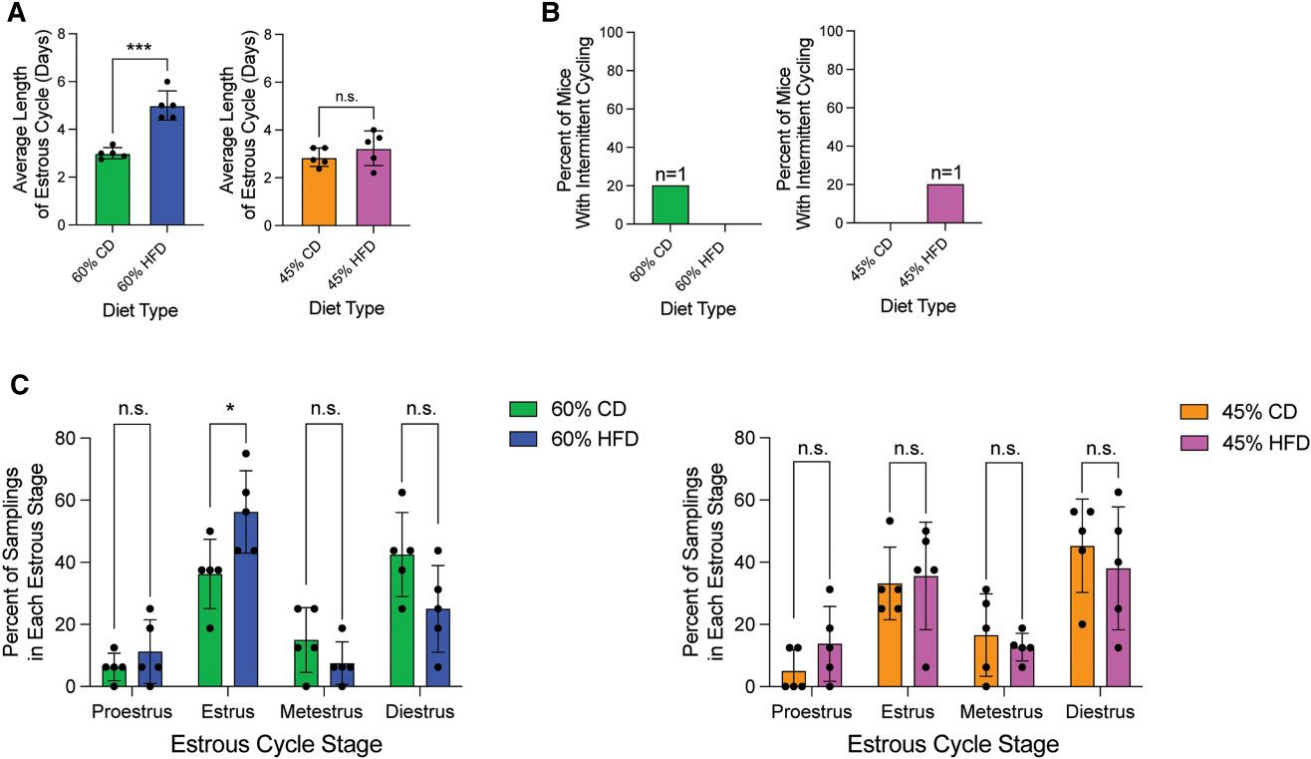
60% HFD alters estrous cycling. (A) Average length of estrous cycle per diet. Measurement (datapoint; n = 5 per condition) represents average cycle length per mouse (bar = diet mean ± SD). Two-tailed unpaired *t*-test statistic. (B) Percent of mice in each diet type that have intermittent estrous cycling (same estrous stage for 6 days or more) over the 2-week lavage period. No statistical analysis was performed. (C) Percent of occurrences in each cycle stage between each HFD and respective CD from the recorded daily lavages (n = 5 per diet). Measurement (datapoint) represents each mouse (bar = mean ± SD). Multiple unpaired *t*-tests statistic with Welch's correction. *P < .05; **P < .01; ***P < .001; ****P < .0001.

Abbreviations: CD, control diet; HFD, high-fat diet.

To determine the effect of HFD on the estrous cycle, starting at 19 weeks on diet, we performed daily vaginal lavage and examined cytology on the mice over a 2-week period. The average estrous cycle length for the 60% HFD mice was significantly longer than the 60% CD, while 45% HFD mice showed no significant difference compared to 45% CD over the 2-week period (Fig. 4A). Of note, there was one 60% CD mouse and one 45% HFD mouse that had intermittent cycling during one of those 2 weeks, as defined by a minimum of 6 consecutive days spent in diestrus (Fig. 4B). Both mice did cycle in either the week before or the week after their pause in cycling though, suggesting a transient cessation of estrous cycling. The prevalence of each estrous cycle stage was compared between each HFD and its respective CD during the 2-week daily lavage time period. Diestrus phase is reported to be the longest estrous cycle stage in standard chow-fed laboratory mice, and in accordance with this, the 45% HFD, 60% CD, and 45% CD mice had the highest mean percentage of samplings in the diestrus stage (38%, 42.5%, and 45.25%, respectively) [21]. In contrast, mice on a 60% HFD had the highest mean percentage of daily samplings in the estrus stage (56.25%), which was also significantly higher than the mean estrus stage sampling percentages for mice on the 60% CD (36.25%). While there was a trend toward 60% HFD having a decreased percent of diestrus stage samplings, it was statistically insignificant from 60% CD. Additionally, the mean percent of proestrus samplings in both 60% HFD and 45% HFD (11.25% and 13.75%, respectively) was roughly doubled when compared to the 60% CD and 45% CD (6.25% and 5%, respectively), though not statistically significant (Fig. 4C).

### Estrous Cycle-Staged 60% HFD Cohort Displays Increased Estrogen-to-Progesterone Ratio and Decreased Corpora Lutea

**Figure 5.**
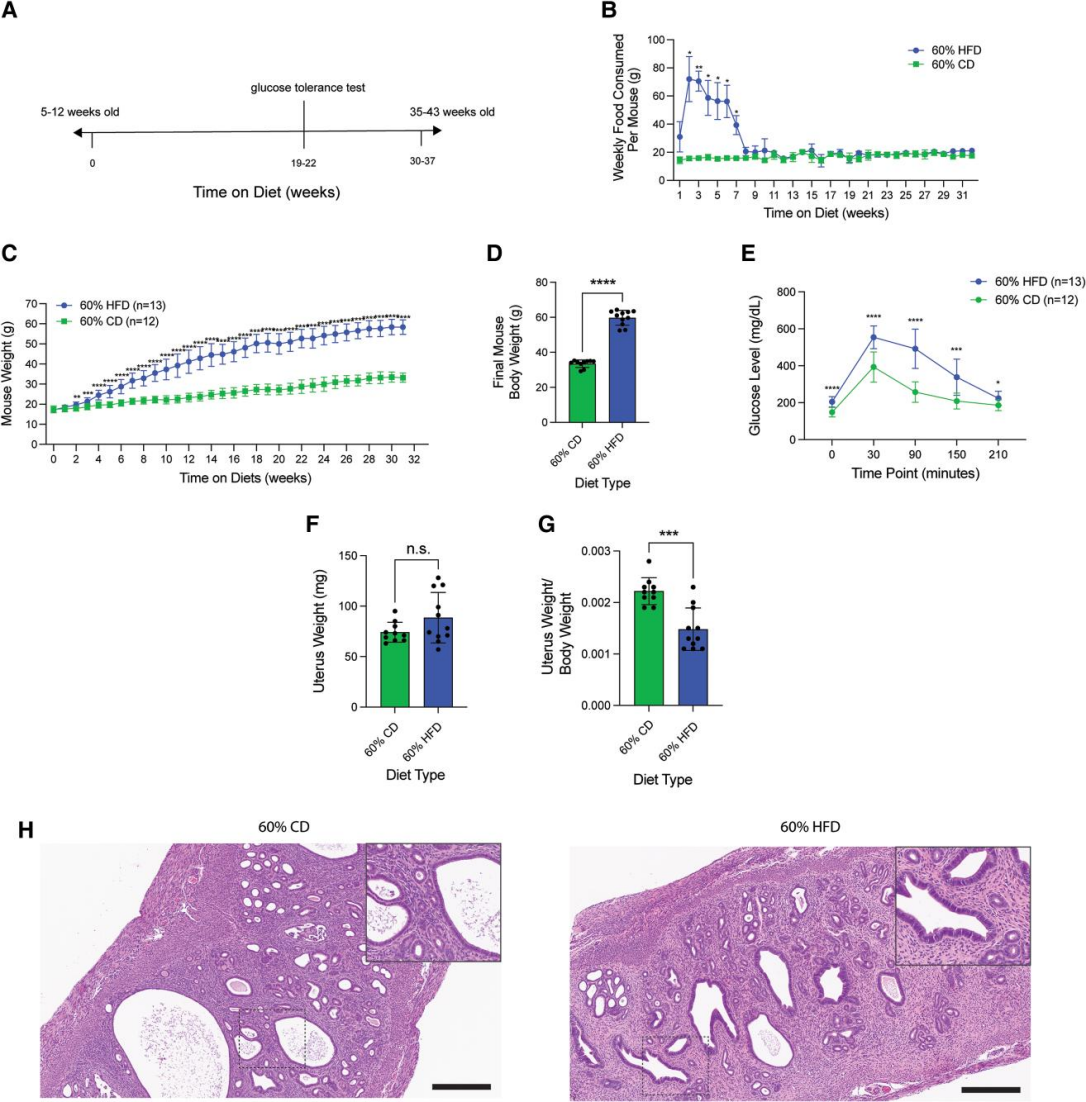
Estrous cycle stage-controlled cohort displays similar obesity-associated metabolic effects from 60% HFD. (A) Experimental timeline for additional 60% HFD and matched-CD cohorts. (B) Estimated average amount of food consumed (grams) per mouse separated per week over the course of the experiment. Measurements represent weekly (datapoint; n = 3 cages per diet type) diet's mean ± SD. Multiple unpaired *t*-tests statistic with Welch's correction. (C) Average mouse body weight (grams) per diet over the time course of the experiment. Measurements represent weekly (datapoint; n = 12 for CD and n = 13 for HFD) diet mean ± SD. Multiple unpaired *t*-tests statistic with Welch's correction. (D) Final mouse body weight (grams) at the time of tissue harvest. Measurement (datapoint; n = 10 for CD and n = 11 for HFD) represents each mouse (bar = mean ± SD.). Two-tailed unpaired *t*-test statistic. (E) Blood glucose measurements (mg/dL) at 30-minute intervals after a 2 g/kg glucose injection of mice at 19 to 22 weeks on diet (n = 12 for CD and n = 13 for HFD). Measurements represent diet's mean ± SD. Multiple unpaired *t*-tests statistic with Welch's correction. (F) Semi-dry uterine weight (mg) at estrus-staged tissue harvest after 30 to 37 weeks on diet. Measurement (datapoint; n = 10 for CD and n = 11 for HFD) represents each mouse (bar = mean ± SD). Two-tailed unpaired *t*-test statistic with Welch's correction. (G) Semi-dry uterine weight to mouse final body weight ratio. Measurement (datapoint; n = 10 for CD and n = 11 for HFD) represents each mouse (bar = mean ± SD). Two-tailed unpaired *t*-test statistic. (H) Representative H&E stain of mouse endometrial tissue for each diet type during estrus. Main image = 10 × magnification; inset = 20 × magnification; scale bar = 300 µm. *P < .05; **P < .01; ***P < .001; ****P < .0001.

Abbreviations: CD, control diet; H&E, hematoxylin and eosin; HFD, high-fat diet.

**Figure 6.**
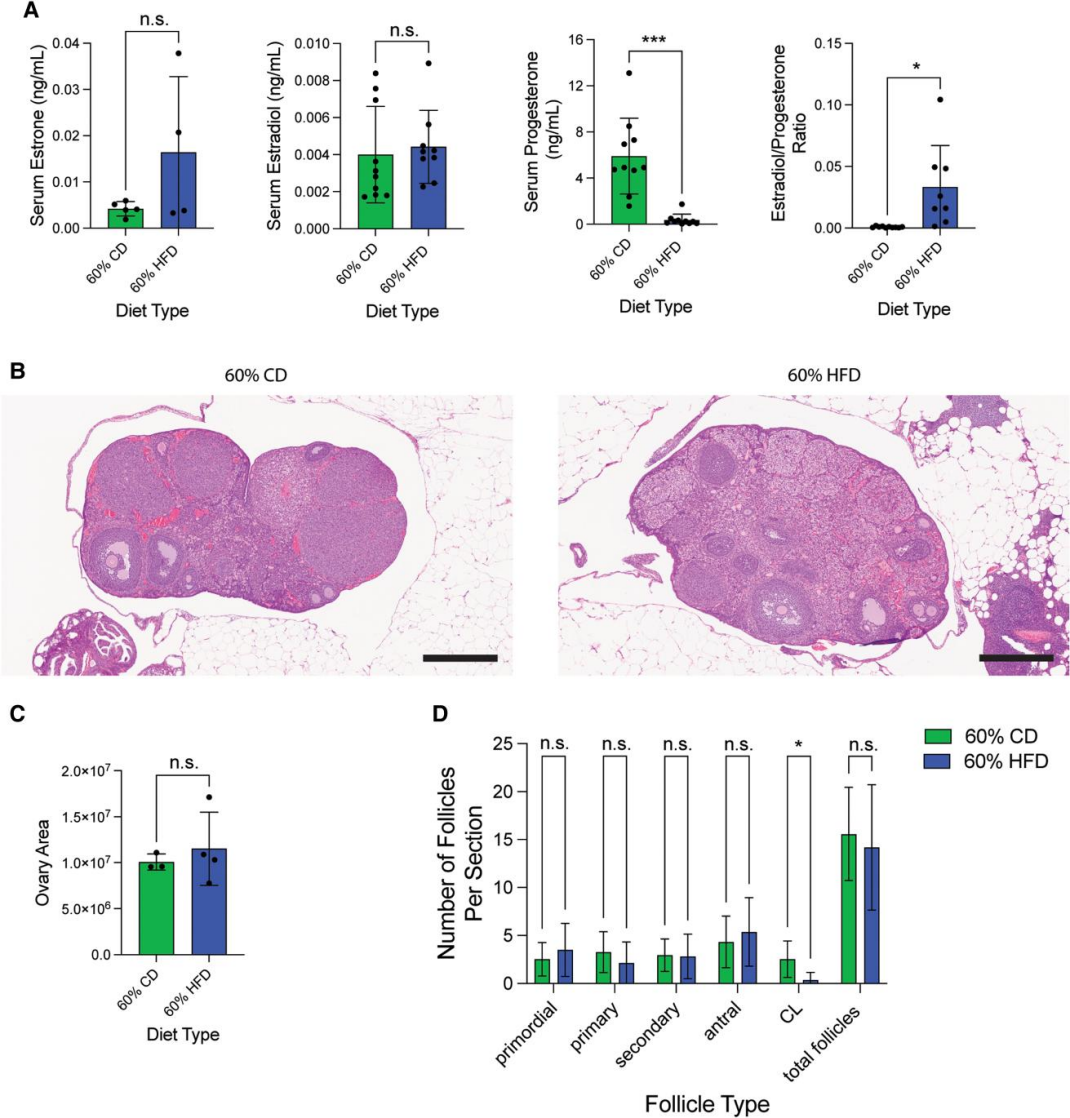
Estrous cycle stage-controlled mice on 60% HFD display unopposed estrogen and decreased corpora lutea (A) Serum estrone (datapoint; n = 5 for CD; n = 4 for HFD), estradiol (datapoint; n = 10 for CD; n = 9 for HFD), progesterone (datapoint; n = 10 for CD; n = 10 for HFD), and estradiol-to-progesterone ratio (datapoint; n = 10 for CD; n = 8 for HFD) levels (ng/mL) from estrus-staged mice after 30 to 37 weeks on either 60% HFD or matched-CD diet. Measurement (datapoint; total measured is n = 10 for CD and n = 11 for HFD) represents each mouse (bar = mean ± SD). Two-tailed unpaired *t*-test statistic for estradiol, and two-tailed unpaired *t*-test statistic with Welch's correction for estrone, progesterone, and estradiol-to-progesterone ratio. (B) Representative H&E stain of mouse estrus-staged ovarian tissue for each diet type. 5 × magnification; scale bar = 500 µm. (C) Largest histological ovarian area for each mouse after 30 to 37 weeks on diet. Measurement (datapoint; n = 4 for CD; n = 3 for HFD) represents each mouse (bar = mean ± SD). Two-tailed unpaired *t*-test statistic. (D) Average number of different follicle types per histological section in each diet-treatment type (n = 19 slides for CD; n = 22 slides for HFD). Bar = mean ± SD. Two-tailed unpaired *t*-test statistic for all follicle types except CL. Two-tailed unpaired t-test statistic with Welch's correction for CL. *P < .05; **P < .01; ***P < .001; ****P < .0001.

Abbreviations: CD, control diet; CL, corpora lutea; H&E, hematoxylin and eosin; HFD, high-fat diet.

**Figure 7.**
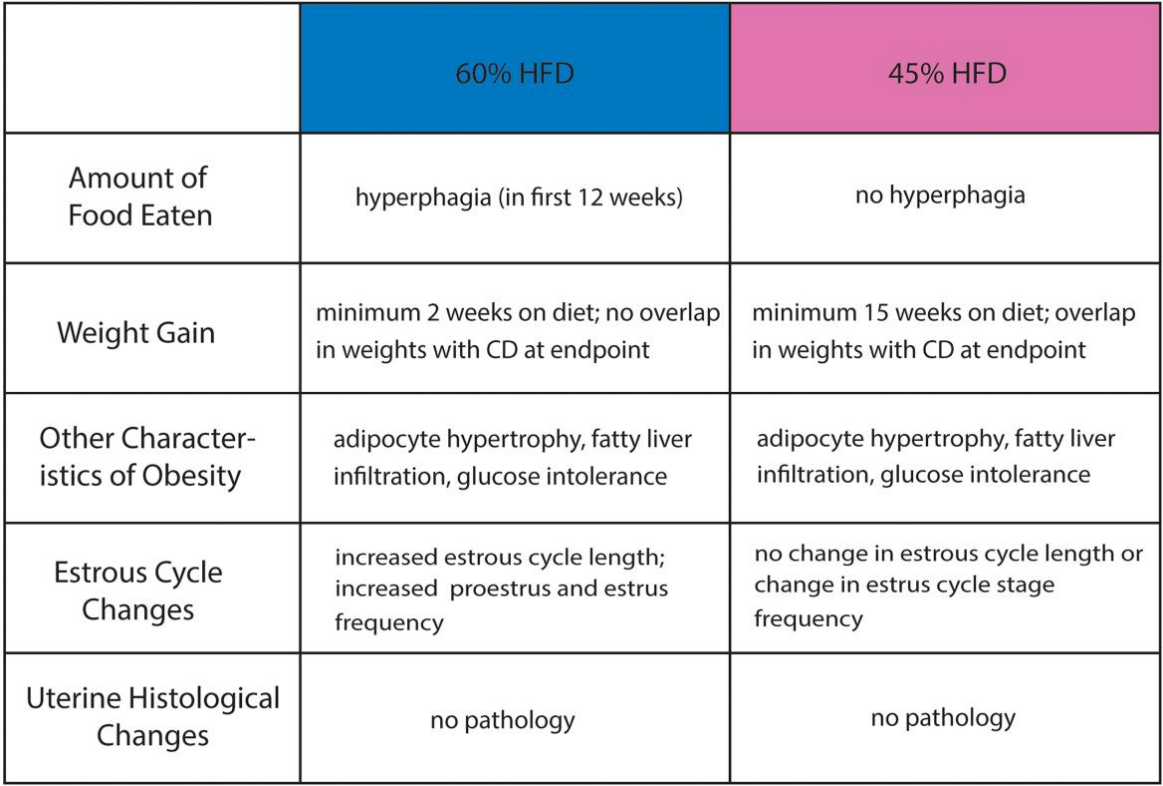
Summary of physiological effects from 60% and 45% HFD in female C57BL/6J mice. Table summarizing the similarities and differences of several general obesity-associated and reproductive tract-specific changes occurring in C57BL/6J female mice in response to 60% HFD and 45% HFD (when each compared to their respective CD).

Abbreviations: CD, control diet; HFD, high-fat diet.

Due to the estrous cycling differences present in 60% HFD-treated mice compared to CD-treated mice, we utilized an additional estrous cycle staged mouse cohort to determine if circulating hormone levels and ovarian histology were also altered (Fig 5A). This second cohort displayed similar patterns of food consumption, weight gain, and glucose intolerance compared to the initial cohort, although this cohort had an elevated fasting glucose level, which was not observed in the first cohort (Fig. 5B-E). After 30 to 37 weeks on the diet, blood, uterus, and ovaries were harvested. Semi-dry uterine weight was unchanged by diet type, but uterus-to-body weight ratio decreased with HFD (Fig. 5F-G). Histological analysis of uterine H&E staining showed no obvious phenotype with HFD, similar to what was observed with the previous cohort (Fig. 5H). Blood was collected for serum to analyze circulating ovarian hormone levels. There were some mice that had undetectable levels of one or more hormones. With the detectable hormone levels we obtained, both circulating estrone and estradiol were unchanged between HFD- and CD-treated animals. In contrast, progesterone levels were significantly decreased in the 60% HFD-treated animals. When calculating hormone ratios, there was a significant increase in estradiol relative to progesterone in HFD-treated mice (Fig. 6A). Since most estradiol and progesterone are produced by the ovary, we next surveyed the ovaries with H&E staining and analysis (Fig 6B). The average ovarian area was similar between HFD- and CD-treated animals (Fig 6C). When examining the amount of each ovarian follicle type, there were fewer CL observed with HFD, which is consistent with CL being a major source of progesterone (Fig. 6D). In summary, we provide comparison data for two HFD treatments in relation to their respective nutrient-matched control diets (CD) in nulligravid female mice, which show differences in amount of food consumed, speed and intensity of weight gain, and estrous cycle alterations (Fig. 7). Upon further investigation into the 60% HFD estrous cycle alterations, we discovered that while 60% HFD-treatment mice maintained a grossly normal uterine phenotype, 60% HFD led to a state of unopposed circulating estrogen and a significant reduction of ovarian CL.

## Discussion

Female mouse models of obesity are needed due to the increasing prevalence of obesity and obesity-associated female reproductive tract abnormalities and pathologies, including infertility, endometrial hyperplasia, and endometrial cancer. Most studies comparing the effects of various DIO methods, do so solely with male mice. It is problematic to assume that female mice respond similarly, as it has been shown that C57BL/6J female mice gain weight slower and have increased glucose tolerance compared to their male counterparts [17-19]. While there is a body of studies utilizing female mice, not all use a properly controlled CD of purified ingredients, minimized phytoestrogens, and matching macro- and micronutrient levels [32, 33]. Therefore, in this study we aimed to test the effects of both a 45% and a 60% HFD on obesity-associated comorbidities, uterine histology, and cyclicity in female C57BL/6J mice compared to their carefully controlled CD. As expected, the 60% HFD mice gained weight faster (after only 2 weeks on diet) and to a larger extent (32.12 g gained in total) compared to 45% HFD (after 15 weeks on diet and a total of 15.79 g). This rapid increase in 60% HFD mouse weight might be partially explained by the hyperphagia seen in the first half of the experiment, which was absent in the 45% HFD mice. Therefore, when picking between the two diets, it is important to consider the rate and magnitude of weight gain desired for a given experiment. Both HFDs produced mice with adipocyte hypertrophy, fatty liver infiltration, and glucose intolerance, though a more severe phenotype was seen with 60% HFD. When comparing these results to our previous work utilizing 60% HFD on outbred CD-1 female mice, the C57BL/6J mice displayed faster and more consistent weight gain and prolonged levels of elevated glucose compared to CD-1 mice [34].

There is debate in the literature over the optimal diet to induce obesity. In obesity-prone C57BL/6 mice, a high-carbohydrate diet in the absence of high fat does not cause an increase in body weight, fat mass, fasting glucose, or insulin [35, 36]. Conversely, HFD alone in C57BL/6 mice causes significant body and adipose tissue weight gain, as well as an increase in fasting glucose and insulin, with or without the inclusion of high sucrose [35]. A standard mouse chow has around 6% to 10% fat, while commonly used HFDs have 45% or 60% fat [10, 11]. A typical American diet consists of 36% to 40% fat, while Americans with obesity might ingest around 50% to 60% fat [10]. Therefore, even though the mouse 60% HFD matches the percentage of fat a person with obesity might ingest, the change in fat from CD to HFD is drastically larger in mice than in Americans without and with obesity [10]. This larger difference in fat might mean that the 60% HFD-treated mice represent the extreme or morbidly obese population in humans. Despite these variations, researchers often use the 60% HFD for DIO studies, due to its quicker weight gain and cost effectiveness [10, 19].

There is controversy regarding using high-fat diet to induce obesity rather than nutritionally mimicking human Western diet-induced obesity. Typical Western diets (TWD) have been developed to model both macro- and micronutrient Western intakes. The TWD has lower amounts of carbohydrates and protein, two-times more fat, fewer micronutrients like calcium and vitamin D, as well as more sodium compared to the AIN93G standard mouse chow [37, 38]. C57BL/6J mice fed the TWD did not gain weight, nor did they develop glucose intolerance or elevated leptin levels compared to the AIN93G control diet mice, while 45% HFD-treated mice did [12]. C57BL/6 mice historically show indifference for sodium consumption compared to other commonly used strains, causing mice to consume less with this diet and, therefore, TWD does not cause significant weight gain in C57BL/6 mice [39, 40]. Common rodent chows contain optimal macro- and micronutrients for rodent health, which are higher in carbohydrates and lower in fats than is recommended for humans [37, 41, 42]. Even among micronutrients, there are different suggested average daily intake levels between rodents and humans, with rodents requiring significantly more calcium [43]. Differing metabolic rates between humans and rodents require altered nutrient profiles, which can make selecting a translational diet challenging [37]. To reduce confounding factors for weight gain, the CD should be closely nutritionally matched to the HFD (excluding the fat content), since micronutrient changes alone can cause weight alterations [12-14]. Food should also be purified vs grain-fed to help maintain diet purity and consistency and minimize phytoestrogens, which are known to influence body weight, adipose deposition, and estradiol levels [14-16].

Genetically-induced obesity models, including *ob/ob* and *db/db* mice, are also widely used to study the effects of obesity in mice. *ob/ob* mice fail to produce leptin, the adipocyte-originating satiety hormone, while *db/db* mice lack the long-form of leptin's receptor (Ob-Rb), which is highly expressed in the hypothalamus and is vital for regulating body weight [8, 44]. Both mouse models exhibit extreme obesity, with weight gain beginning as early as 4 to 5 weeks of age, and lead to a 4-fold total increase in body weight. These obese mice additionally develop obesity-associated comorbidities including hyperglycemia and hyperinsulinemia [45-48]. This model of obesity lacks translational relevance, as it is rare to see either of these genetic mutations in humans with obesity [7, 9, 49]. Instead, individuals with obesity have high levels of leptin, so it is thought that humans with obesity are leptin resistant due to the prolonged elevated leptin levels [50-52]. Moreover, when studying obesity's impact on the female reproductive tract or fertility, the *ob/ob* and *db/db* models of obesity can be problematic, as these mouse models exhibit decreased uterine size and impaired fertility [48, 53-55]. The infertility seen with these *ob/ob* and *db/db* mice can then make it difficult to breed additional mutant alleles into this line.

In addition to different diets, there is also a variety of mouse strains used to study HFD-induced obese phenotypes. Previous data published in our lab saw phenotypic variability in HFD-induced obese mice using an outbred CD-1 strain [34]. While the CD-1 mice exhibited obesity, glucose intolerance, and hyperinsulinemia, an outbred strain may hide differences between the HFD and CD animals due to genetic variability [34]. To better identify the impacts of obesity, it might be beneficial to use an inbred line like the C57BL/6J to reduce the genetic variability. C57BL/6J mice consistently exhibit obesity with HFD [11, 35, 52, 56, 57]. Like humans with obesity, C57BL/6J mice on a HFD also show an increase in circulating leptin and decreased leptin signaling, hyperinsulinemia, and hyperglycemia [35, 52, 57, 58]. AKR/J mice also display an obese phenotype on HFD, by an increase in body weight, hyperinsulinemia, and insulin resistance [59-61]. Despite the similarities between these strains, C57BL/6J mice present with a significantly stronger glucose intolerance, while the AKR/J exhibit a greater resistance to insulin [59]. BALB/c mice have increased glucose tolerance on HFD compared to C57BL/6J [17]. A/J mice display a significant but weaker obesity phenotype on HFD compared to C57BL/6 with regard to the amount of body weight gain, increase in adiposity, fasting glucose, and hyperinsulinemia [35, 57]. Even among C57 strains, the C57BLKS/J strain has a weaker obesity phenotype than the C57BL/6J mice, as the BLKS/J retain normal insulin levels on HFD [62].

Females with obesity, defined by increased waist circumference, are known to have longer menstrual cycles [5]. This increase in cycle length was reflected in our 60% HFD-treated mice. When looking at the individual stages of the normal estrous cycle in a chow-fed wild-type mouse, the greatest amount of time is typically spent in diestrus [21]. This was not the case with the 60% HFD-fed mice, which had the highest percent of samplings in estrus (56.25%). Moreover, the 60% HFD had roughly double the percent of samplings in proestrus when compared to their CDs. This increase in proestrus and estrus stages might be due to the elevated levels of estrogen relative to progesterone, which are observed in women with obesity [3, 63, 64]. Mouse estrogen levels are highest in the proestrus stage and start to decline as mice progress through estrus [65]. Our results conflict with findings in Hua et al (2020), who also found an increase in the length of cycles in 60% HFD C57BL/6J female mice, but observed an increase in the amount of time spent in diestrus compared to CD mice [33]. Conversely, Lenert et al (2021) found that 45% HFD C57BL/6J mice had a higher percentage of samplings in proestrus and estrus compared to CD, though this occurred after short exposure to HFD, as the study examined timepoints before the mice developed obesity [32]. The estrous cycle can also impact behaviors such as the stress response and response to male-emitted pheromones [66, 67]. Therefore, the impact of HFD on estrous cycling should be kept in mind when performing studies on female mice, even if the female reproductive tract is not being studied.

The majority of estrogen and progesterone in premenopausal nongravid women are produced in the ovary [3, 63, 68, 69]. While we found normal levels of estradiol, we found that progesterone levels were significantly decreased in HFD-treated animals, leading to a state of unopposed estrogen. In accordance with this hormone imbalance, we found that the ovaries of 60% HFD-treated mice contained a decreased number of CL, which is the primary site of progesterone synthesis [70]. This imbalance of a high estrogen-to-progesterone ratio could increase susceptibility to the estrogen-dominated proestrus and estrus stages observed in 60% HFD-treated mice. Studies have shown that women with obesity spend a longer amount of time in the proliferative phase (estradiol-dominant) or a shorter amount of time in the luteal phase (progesterone-dominant) of the ovarian cycle [71, 72]. Estrogen unopposed by progesterone is associated with an increased risk of endometrial hyperplasia, since endometrial epithelial proliferation is stimulated by estrogen [73-75]. Therefore, elevated estrogen levels in women with obesity corresponds with their 3-fold increased risk of endometrial hyperplasia [63, 76]. HFD induced no obvious uterine histopathology by H&E or KRT8 staining, even when controlling for estrous cycle stage. This might be explained due to our HFD mouse model still showing low, but detectable, progesterone levels. It is possible that longer-term HFD studies would lead to uterine pathologies in mice.

One limitation of our study is that the obesity-stimulating “Western diet” nutrient profile was not used to stimulate obesity in this study due to its known insufficiency of C57BL/6J mouse weight gain [12]. Therefore, the two HFDs described here might not be useful when studying a specific obesity-associated metabolite. Another limitation of the study is that we chose to collect samples during the estrus stage due to its increased prevalence with 60% HFD. This stage-specific collection could mask findings present at other cycle stages, but to avoid variability in the data, both HFD and CD-treated mice should be collected at the same stage. Lastly, it is important to note that while we performed vaginal lavage every 24 hours to determine estrous cyclicity, mice on average stay in the proestrus and metestrus stages for less than a day [21]. Since proestrus and metestrus cycle stages can be less than 24 hours in length, sampling multiple times a day may yield a more robust estrous cycle profile.

In summary, this study examines the effect of diet-induced obesity on C57BL/6J obesity-associated comorbidities, uterine histology, and cyclicity using two HFD-induced obesity treatments when compared to their respective sucrose and nutrient-matched CDs. Both 45% and 60% HFD stimulated significant weight gain, adipocyte hypertrophy, fatty liver infiltration, and glucose intolerance and lacked a uterine histological phenotype. However, only 60% HFD induced hyperphagia, as well as an increased estrous cycle length and estrus stage frequency. When examined further, 60% HFD-treated mice showed an increased ratio of estradiol to progesterone and a decrease in ovarian CL compared to CD. Therefore, the differing effects of 45% and 60% HFD should be considered when selecting a HFD for study of the female reproductive tract, but may not be relevant to the study of other organ systems, since obesity-associated metabolic alterations were present in both HFDs. Additionally, if 45% HFD-treated mice were kept on the diet for longer, they might eventually show reproductive alterations like those observed in 60% HFD-treated mice. With these results in mind, care should be taken when planning and executing HFD-induced obesity studies utilizing female mice.

## Data Availability

Original data generated and analyzed during this study are included in this published article or in the data repositories listed in References.
